# Recent advances in electrochemical sensor technologies for THC detection—a narrative review

**DOI:** 10.1186/s42238-022-00122-3

**Published:** 2022-03-15

**Authors:** Kaveh Amini, Ali Sepehrifard, Ali Valinasabpouri, Jennifer Safruk, Davide Angelone, Tiago de Campos Lourenco

**Affiliations:** Selective Lab Inc., Richmond Hill, ON Canada

**Keywords:** Δ^9^-tetrahydrocannabinol, THC, Sensor, Cannabis, Electrochemical detection

## Abstract

**Background:**

Δ^9^-tetrahydrocannabinol (THC) is the main psychoactive component and one of the most important medicinal compounds in cannabis. Whether in human body fluids and breath or in laboratory and field samples, rapid and easy detection of THC is crucial. It provides insights into the impact of THC on human organism and its medicinal benefits, it guides the cannabis growers to determine different stages of the growth of the plant in the field, and eventually it helps scientists in the laboratory to assure the quality of the products and determine their potency or better understand the product development procedures. The significance of fast THC detection in forensic analysis also cannot be overlooked. Electrochemical sensor technologies are currently in the focus of attention for fast, easy, and low-cost detection of THC.

**Method:**

In this work, we review the recent advances in sensor technologies developed for the purpose of fast and accurate THC detection. The research works performed mostly in the past decade and those detecting THC directly without any derivatization were the main target of this review. The scope of this narrative review was the reports on detecting THC in synthetic samples and plants as well as oral fluid.

**Results:**

Electrochemical sensor technologies are sensitive enough and have the potential for fast, easy, and low-cost detection of THC for roadside testing, THC trending in growing cannabis plants, THC product development and formulation for medical purposes, etc., and they can provide an alternative for costly chromatography and mass spectrometry-based methods.

**Conclusion:**

The main challenges facing these sensors, however, are nonspecific interaction and the interference of compounds and species from the matrix. Special requirement for storing sensors modified with antibodies or proteins is another challenge in this field. Preparing long-lasting and reusable sensors is a field worthy of attention.

## Introduction

In the light of legalization of medicinal and recreational cannabis in Canada and many states in the USA as well as other countries around the world, a great deal of attention has been given to the research in different areas of cannabis chemistry (Crean et al. 2011). In addition, the evidence for therapeutic effect of cannabis has turned cannabis research into a hot topic (Hill 2015; Hoffmann and Weber 2010). Over 60 unique cannabinoids have been identified in the cannabis plant (Vemuri and Makriyannis 2015). Out of these cannabinoids, Δ^9^-tetrahydrocannabinol (THC), cannabidiol (CBD) and cannabinol (CBN) are the most significant ones (Grotenhermen 2003). THC is responsible for the psychoactive property of cannabis and causes euphoria, drowsiness, hallucinations, and temporal distortions (Ashton 2001). CBD, another significant cannabinoid, on the other hand is not psychoactive; however, it has neuroprotective, sedating, anti-inflammatory, and analgesic impacts (Chakravarti et al. 2014; Hill 2019; Klimuntowski et al. 2020; Mechoulam et al. 2007). The legalization of cannabis also has raised concerns about driving under the influence of THC. An increase in the number of cases of THC-impaired driving also has been reported in the regions where cannabis has been legalized (Kalant 2001; Zuardi 2006; Kim et al. 2013).

**Fig. 1:**
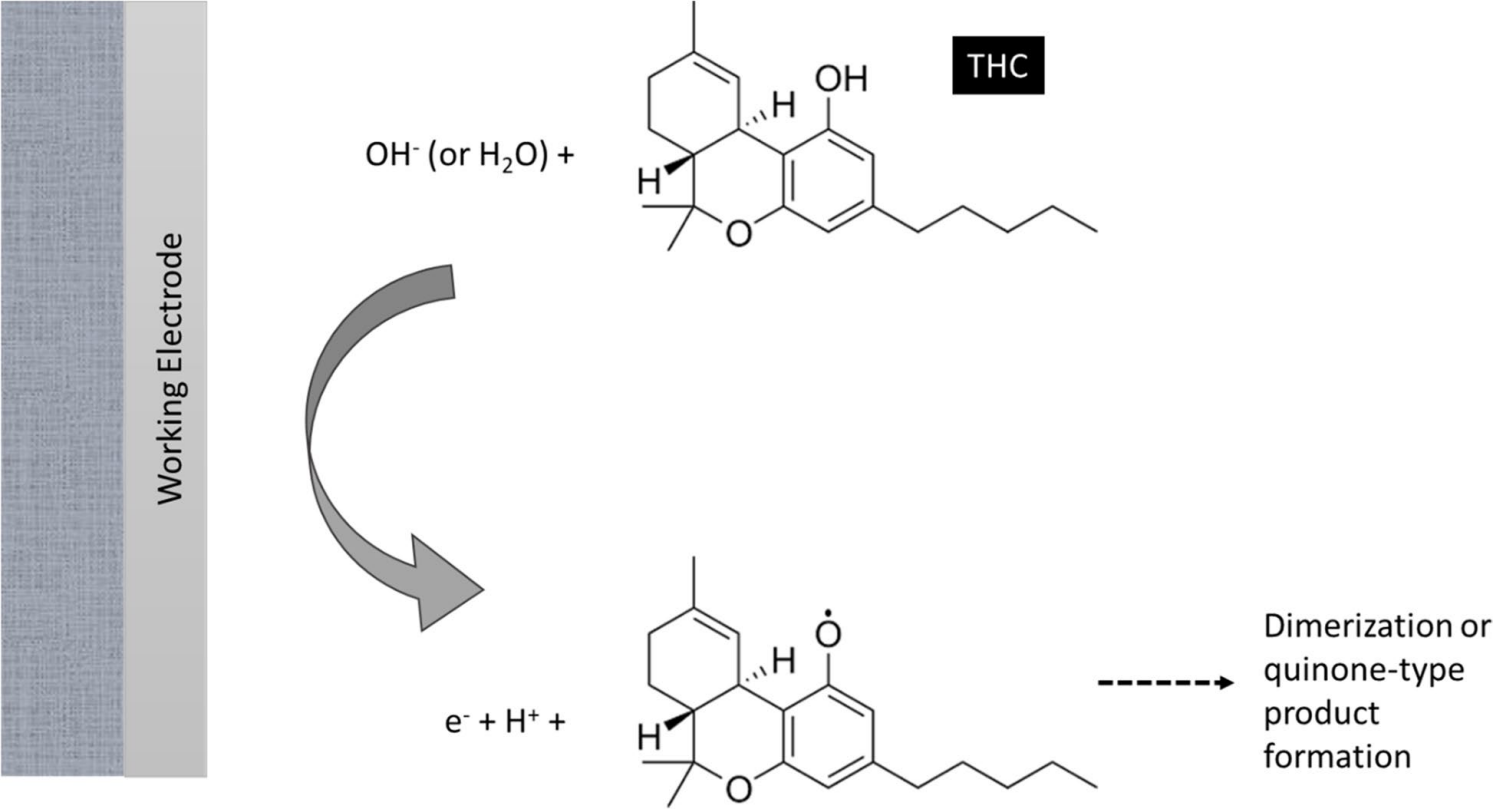
Mechanism of detection of THC on an electrochemical sensor. The illustrated mechanism is according to (Renaud-Young et al. 2019)

Electrochemical sensors provide a highly sensitive tool for the analysis of THC, and they also have advantages such as easy miniaturization and usability in turbid matrices. In these sensors, the current or the potential changes as a result of the interactions or reactions at the interface between the sensor surface and the sample solution is measured. On the basis of the electrochemical technique used for detection, these sensors are categorized as impedimetric, amperometric, voltammetric, and potentiometric. In Impedance-based sensors, electrochemical impedance spectroscopy (EIS) is employed as the detection technique. In these sensors, a low voltage sinusoidal potential is applied at different frequencies to the sensor and the impedance is measured as a function of frequencies using the resulting current. The interaction between the analyte and a biorecognition element immobilized on the sensor surface will cause changes in the impedance. The results will be interpreted in terms of an equivalent circuit. The major advantage of impedance-based sensors is being label-free. In the case of amperometric sensors, the changes in current are followed. Voltammetric sensors employ electrochemical techniques such as cyclic voltammetry, square wave voltammetry, and differential pulse voltammetry. In potentiometric sensors, measurements are based on development of electrochemical potential in proportion to the activity of the analyte (Amini and Kraatz 2015a, b). Figure 1 illustrates the mechanism under which THC electrochemical sensors operate according to (Renaud-Young et al. 2019).

The standard THC detection method includes costly, complex, and time-consuming steps of obtaining a blood sample and analyzing it using chromatography with mass spectrometry detection. This method obviously would be on the back foot when it comes to in-field and roadside testing. These limitations of standard THC detection methods have motivated the scientists to develop portable, non-invasive, fast, and low-cost sensor technologies for on-site THC screening testing (Sivashanmugan et al. 2019). Sensors capable of detecting and quantifying THC and other cannabinoids also can be very effective in other areas such as monitoring different stages of plant growth, quality control of cannabis products, and during product development from cannabis. Also, the ease of use alongside rapid detection by these sensors makes them ideal to be used by dispensaries or consumers to evaluate the THC levels or THC-CBD ratio of the products (Comeau et al. 2019). Sensor technologies with great reduction in analysis time and cost as well as better sensitivity and possibility of miniaturization in comparison to other portable methods offer a novel and alternative approach (Amini and Kraatz 2015a, b; Amini and Kraatz 2016; Jadon et al. 2016) to testing cannabinoids in particular THC, for different purposes. In this work, we critically review the recent developments and advances in sensor technologies for THC detection and the potential presented for different applications from forensic and law enforcement to quality control and product development. The sensors will be discussed in two major categories: (a) synthetic solutions and plant analysis and (b) oral fluid analysis. Sensors focused on THC detection in breath which are thoroughly reviewed by Ramzy and Priefer (Ramzy and Priefer 2021) have not been included in this review.

- Development of early sensors and trials in synthetic solutions and plant extracts

**Fig. 2:**
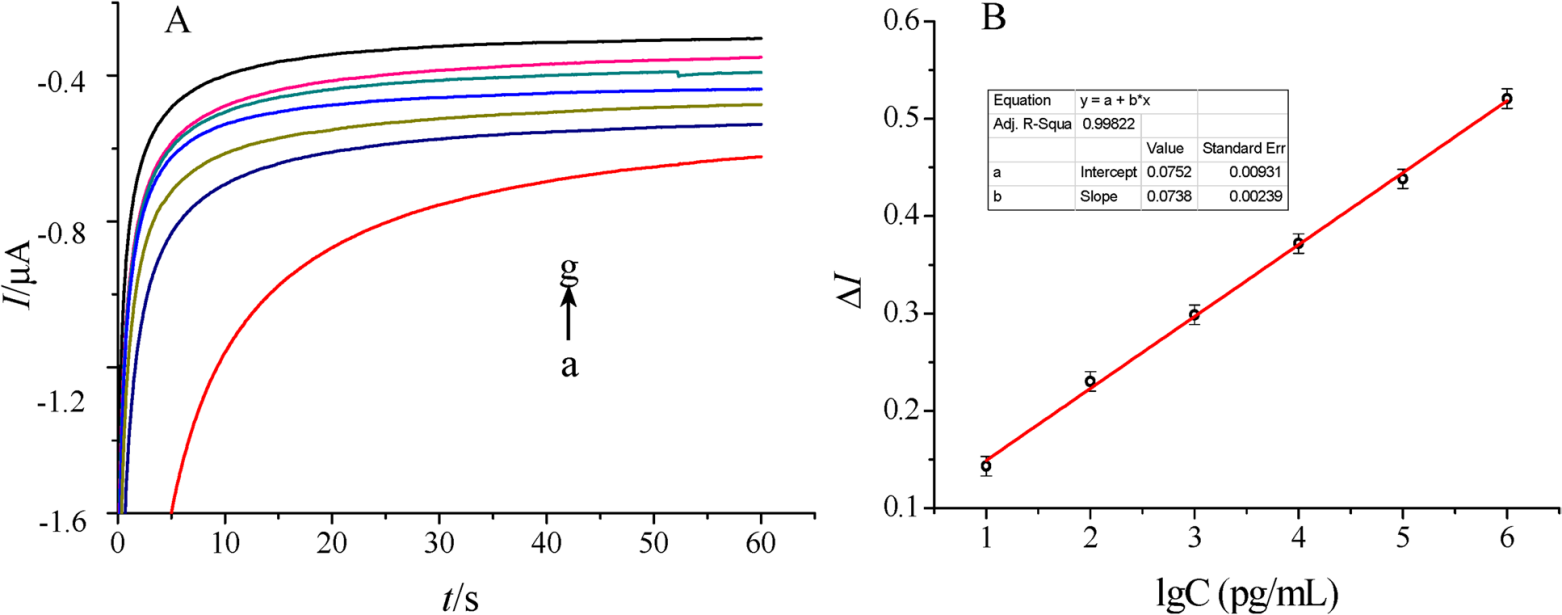
**A** Current response curves obtained for the determination of THC: a 0.01 M PBS (phosphate buffer saline) solution at pH 7.4 as the blank, b~g are the signals obtained for diluted THC solutions at increasing proportions with PBS, the mass concentration has been 0.01~103 ng/mL. **B** Calibration curve for the determination of THC. Taken from (Dingqiang et al. 2016) under permission policy of Molecules (Copyright 2016)

**Fig. 3:**
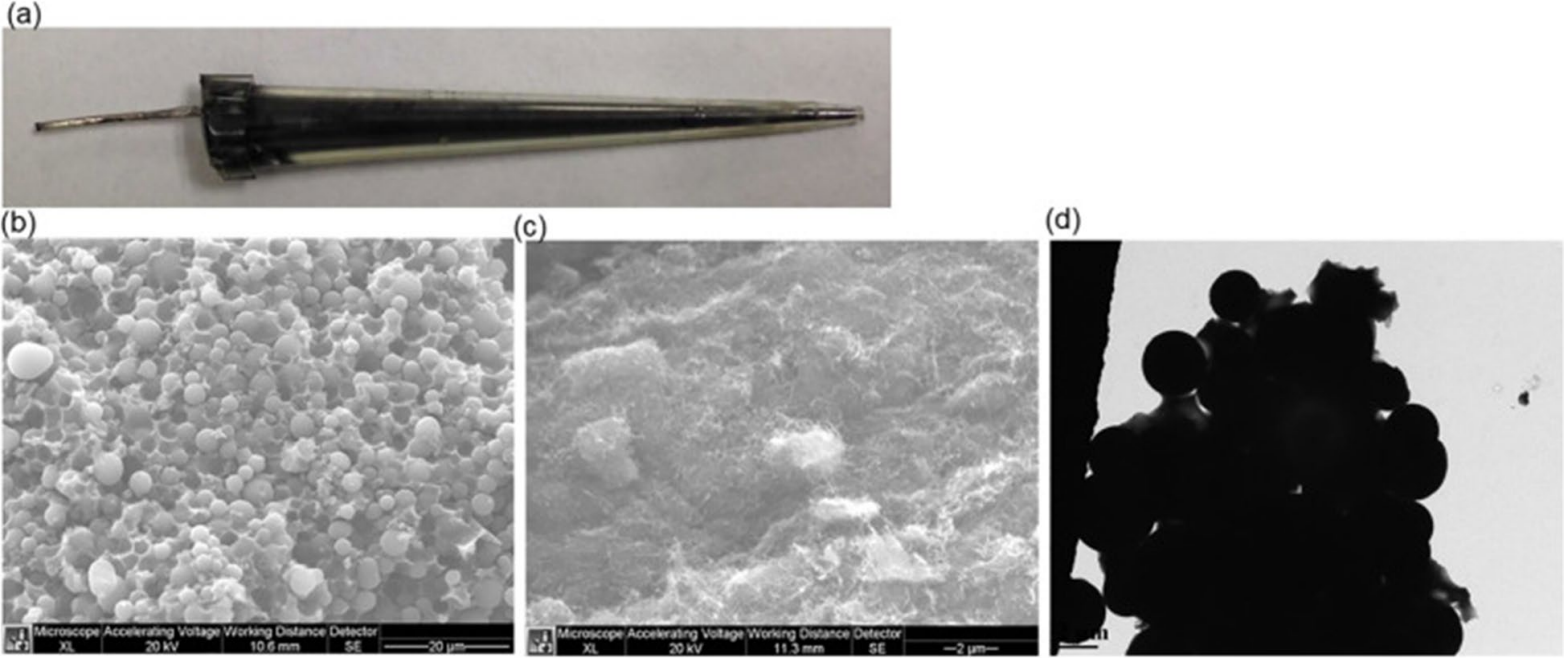
**a** Optical image of a carbon beads-based sensor. **b** SEM (scanning electron microscope) images of carbon beads-based sensor. **c** CNT (carbon nanotube)-based sensor. **d** TEM (transmission electron microscope) image of carbon beads based sensor. Taken from (Zhang et al. 2019) with permission from Elsevier (Copyright 2019)

The first attempt for the development of a THC sensor has been reported by (Dingqiang et al. 2016). To develop this sensor, a tetrahydrocannabinol (THC) antibody derived from Balb/c mice as the bio-recognition element has been immobilized on a novel double-layer gold nanoparticles electrode. Also, an electrochemical biosensing signal amplification system with gold nanoparticles-thionine-chitosan absorbing horseradish peroxidase (HRP) has been used to enhance the number of immobilized antibodies and thus the electrochemical signal. Developed biosensors have been employed to determine THC in phosphate buffer saline (PBS) using the amperometric I-t curve method. The dynamic linear range of the calibration curve made by plotting response current versus THC concentration has been from 0.01~10^3^ ng/mL with a correlation coefficient of 0.9986. The lowest limit of detection for THC also has been reported to be 3.3 pg/mL (S/N = 3) with good sensitivity and reproducibility. Current response curves for determination of THC and the calibration curve constructed in the work by (Dingqiang et al. 2016) have been illustrated in Fig. 2. More recently, Zhang et al. have employed carbon nanotubes (CNT) or carbon beads and poly(methyl acrylic acid-co-ethylene glycol dimethacrylate) (poly(MAA-Co-EGDMA)) with molecularly imprinting technology in micropipette tubes to develop THC sensors. Molecularly imprinted polymers (MIPs) are synthetic receptors which can selectively bind to their target molecules and, therefore, can be used as recognition elements in sensors as a replacement for relatively unstable bio-recognition elements such as enzymes and antibodies. The report emphasizes on the importance of utilizing carbon materials in sensors which results in a high sensitivity because of the high surface area as well as MIPs technique to create THC molecular cavities on the surface of carbon micro-beads and carbon nanotubes (CNT) packed in plastic micropipette tips. These THC MIPs have been synthesized by copolymerization of methacrylic acid (MAA) and ethylene glycol dimethacrylate (EDGMA) in presence of THC initiated with 4,4′-azobis (4-cyanovaleric Acid) (AIBN) and then THC has been removed to generate THC molecular cavities for specific binding of THC. Optical and microscopic images of the sensors developed have been shown in Fig. 3. The developed sensors have shown high selectivity towards THC over caffeine and acetaminophen. The detection limit of THC using CNT-MIP sensors has been determined to be 0.18 ± 0.02 ng/mL which is significantly improved in comparison to the detection limit using nonimprinted polymers (NIP) which is 12.5 ± 0.5 ng/mL (Zhang et al. 2019). Another work employing MIPs has been introduced by Canfarotta et al. In this report, a manufacturing-friendly protocol for integration of MIP nanoparticles (nanoMIPs) with a (label-free) capacitive sensor has been developed. The two templates for which the nanoMIPs have been produced include THC as a small molecule and trypsin as a protein. Using this sensor, determining THC has been demonstrated to be possible at physiological concentrations. Another advantage of these sensors has been indicated to be the possibility of utilizing these sensors for detection and quantification of other biomolecules by varying the nanoMIPs. These nanoMIPs can be virtually produced against any target (Canfarotta et al. 2018).

(b) Sensors developed for THC detection in oral fluids samples

Oral fluid or saliva analysis for roadside drug testing is a great substitute for routine tests due to its non-invasive nature compared to blood analysis and removing the need for inconvenient observations during sample collection for urine analysis. These tests are normally performed as a screening step. Goodwin et al. have reported a sensor developed by modification of micro-sized graphite powder by abrasive immobilization of 4-amino-2,6-diphenylphenol onto a basal plane pyrolytic graphite electrode. This sensor has been then used for indirect detection of THC in oral fluid. The mechanism under which this sensor operates is that by addition of THC, the reduction wave, which corresponds to the electrochemical reduction of quinoneimine (QI) back to aminophenol (AP), reduces in magnitude due to the reaction between QI and THC, and this, in turn, provides a useful analytical signal. This technique has been indicated to be very attractive because it does not involve direct oxidation of THC which can cause electrode passivation. Goodwin et al. have reported that this work has had the potential to detect THC using screen printed electrodes and similar approaches. The construction of the sensor has been done through machining pyrolytic graphite discs into 4.9 mm diameter. The counter electrode has been a platinum wire and the measurements have been performed against a saturated calomel electrode. The linear dynamic range of the sensor has been reported to be from 1.25 to 25 μM with a limit of detection found to be 1 μM (Goodwin et al. 2006).

**Fig. 4:**
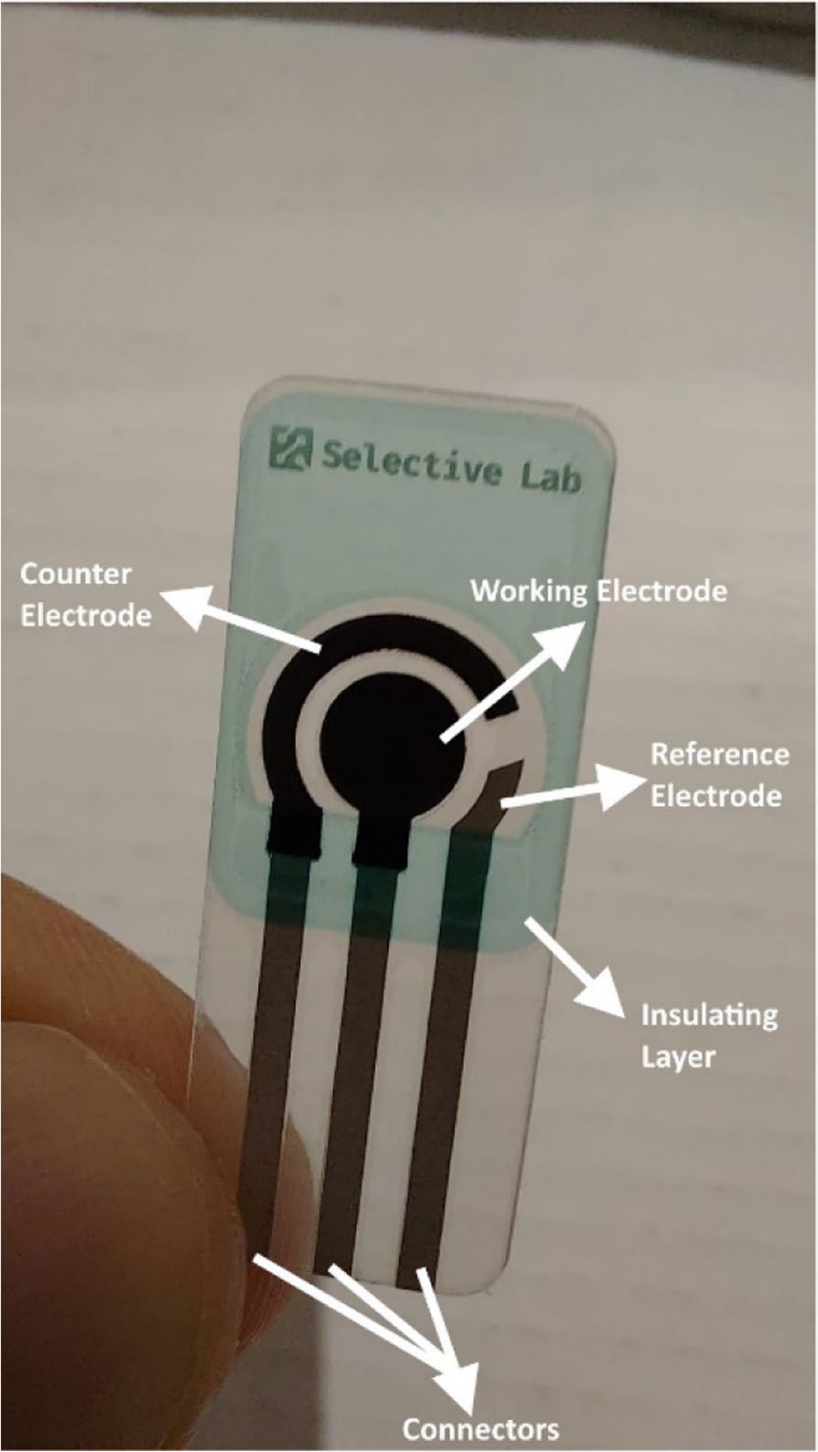
A screen-printed electrode and its different parts

Screen-printed electrodes are produced by using different kinds of inks on various types of plastic or ceramic substrates. A wide variety of these screen-printed electrodes are commercially available. The versatility of screen-printed electrodes is due to wide range of possibilities for the modification of the electrodes. The composition of the inks can be modified by addition of different substances such as metals, enzymes, polymers, complexing agents, etc. Also, it is possible to modify the electrodes by depositing different substances on the surface of the electrodes such as metal films, polymers, enzymes, etc. (Suresh et al. 2021). Figure 4 illustrates a screen-printed electrode and its different parts.

**Fig. 5:**
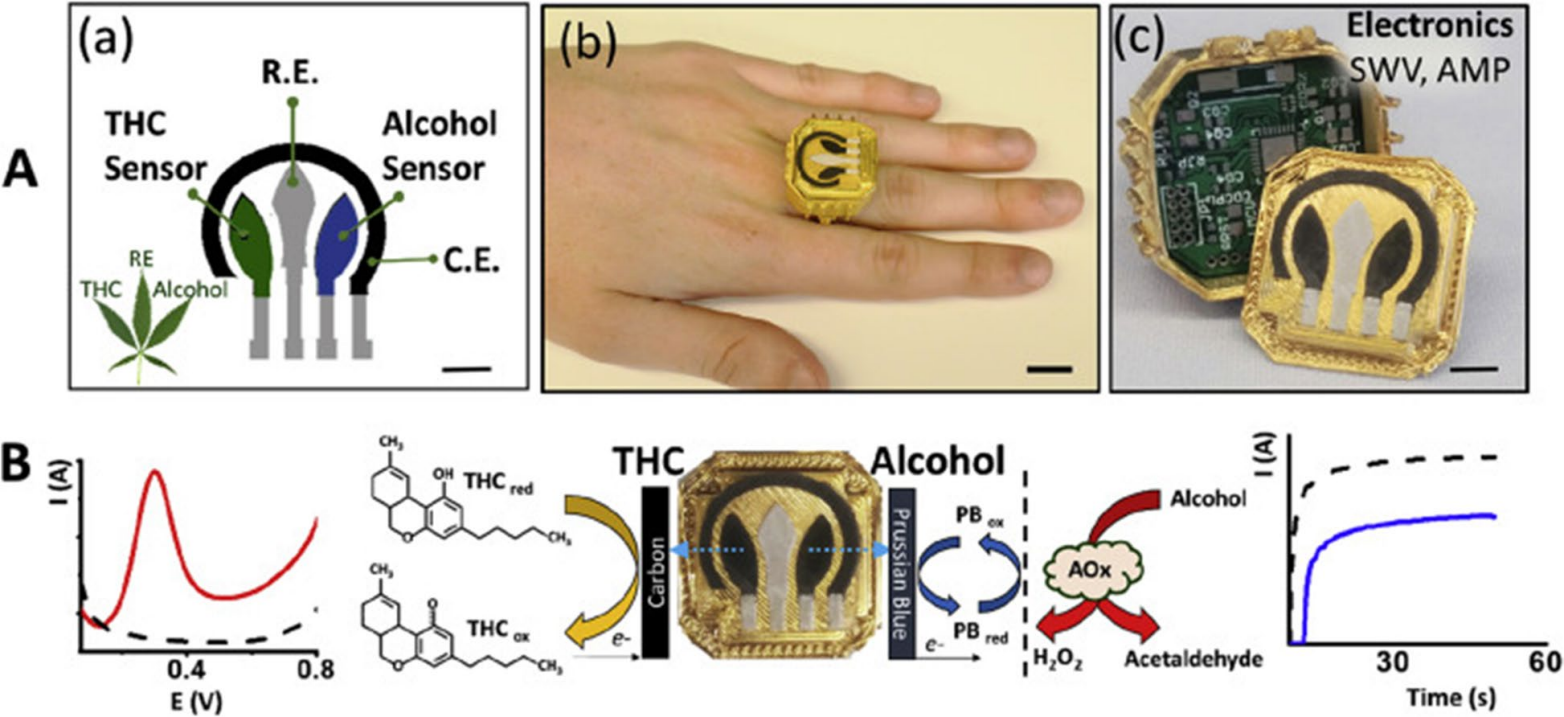
**A** (a) The sensor designed by Mishra et al. for simultaneous detection of THC and alcohol. (b) The image of ring-shaped sensor. (c) The ring polymeric case with the embedded electronics and replaceable screen-printed electrodes section. **B** Mechanisms of THC and alcohol detection using the ring sensor. The voltammogram for the detection of THC (in red) and the amperogram of alcohol detection is also illustrated (in blue). Taken from (Mishra et al. 2020) with permission from Elsevier (Copyright 2020)

In another interesting report, Mishra et al. have presented a work on a wearable electrochemical sensor for the simultaneous direct, decentralized, detection of THC, and alcohol in oral fluids. This sensor has been designed in the form of a ring and includes a voltammetric THC sensor and an amperometric enzymatic alcohol sensor on the ring cap with the wireless electronics embedded within the ring case. The disposable sensing electrode ring cap has been designed so that it allows for fast replacement through alignment with the spring-loaded pins, mounted on the electronic board (PCB), with the current collectors of the sensing electrodes, after each oral fluids test. The printed sensor for dual-analyte detection (ring cover) has been composed of a multi-wall carbon nanotube/carbon electrode for THC detection and a Prussian-blue transducer, coated with alcohol oxidase/chitosan reagent layer, for alcohol detection. This structure makes it possible for THC and alcohol to be detected simultaneously in the same diluted oral fluids sample in 3 min without any interference from the matrix and also no cross talk among sensors. Figure 5 illustrates the ring sensor developed by Mishra et al. The electrochemical technique used for THC and alcohol detection has been square-wave voltammetry and amperometry respectively. The detection limits have been indicted to be 0.5 μM THC and 0.2 mM alcohol. This wearable THC/alcohol sensor has been found promising for both roadside drug testing as well as for drivers’ self-assessment before driving (Mishra et al. 2020).

In another work, Nissim and Compton have introduced an optimized carbon paste electrode, made from graphite powder and mineral oil, for sensitive detection of THC in both aqueous solutions of pH 10.0 and in synthetic oral fluids. Absorptive stripping voltammetry has been utilized as the detection technique. A copper rod with a radius of 1.97 mm running through a Teflon rod has been used to fabricate the carbon paste electrode. The copper rod in Teflon rod has been adjusted so that it leaves 1.00 mm deep cavity at the edge. Two sorts of pastes have been used which have been made by mixing graphite powder with either dioctyl phthalate or mineral oil. 1.4 mL dioctyl phthalate and 4.26 g graphite powder have been mixed to prepare the graphite/dioctyl phthalate paste. The same ratio also has been used to prepare the graphite/mineral oil paste. The surface of the carbon paste electrode has been renewed between each scan by packing fresh paste. Practical limits of detection for THC using this sensor have been reported to be 0.50 μM and 0.10 μM in stationary and stirred aqueous borate buffer solutions, respectively, whereas the theoretical limits of detections have been calculated to be 0.48 nM and 0.41 nM for stationary and stirred THC aqueous borate buffer solutions, respectively. This sensor has been capable of detecting THC concentrations as low as 0.50 μM in synthetic oral fluids solutions. The sensor has had sensitivities of 0.12 μA μM^−1^, 0.84 μA μM^−1^ and 0.067 μA μM^−1^ for the stationary buffer, the stirred buffer, and the oral fluids matrix, respectively (Nissim and Compton 2015).

Wanklyn et al. have also reported development of a screen-printed carbon electrode for N-(4-amino-3-methoxyphenyl)-methane sulfonamide mediated detection of THC in oral fluids. The sensor has been prepared by placing a dried reagent overlayer containing mediator, buffer, salt, and surfactant over the electrodes. When applying the sample to one end of the membrane, the sample would go through the overlayer and wet the reagents and the electrode surfaces and thus dissolving the mediator. The mediator would galvanostatically get oxidized and react with THC to form an electrochemically active adduct which would be in turn detected by chronoamperometric reduction. The developed sensor has been used to detect THC spiked in undiluted oral fluids at 25–50 ng/mL with a response time of 30 s. A trial of these sensors on the oral fluids samples from four cannabis smokers has shown a sensitivity of 28 %, specificity of 99% and accuracy of 52%. The sensitivity of this sensor has been indicated to be lower than the acceptable criteria (Wanklyn et al. 2016).

Stevenson et al. also have introduced an impedance-based sensor utilizing affinity biosensing to detect THC in oral fluid through its chemical reaction with a specific antibody (Stevenson et al. 2019). The detection limit for this sensor has been indicated to be 100 pg/ml and the dynamic linear range has been 100 pg/ml–100 ng/ml in human oral fluid. This sensor has had a rapid detection time, i.e., below 1 min. In this sensor technology, non-faradaic electrochemical impedance spectroscopy is used to detect the presence of BSA-THC hapten in human oral fluid. Affinity biosensor has been shown to detect the biomarker through a recognition element that specifically binds to the biomarker of interest. The chemical reaction between the biomarker and the recognition element has then been converted into an electrical signal correlated to the concentration of the biomarker. This electrochemical biosensor has been capable of streamlining the testing process, removing the need for sample preparation and reducing the analysis time.

## Conclusion and future perspectives

There is a great amount of focus by researchers around the world on the development of a hand-held, fast, and user-friendly device for the detection of THC for different applications of roadside drug testing, cannabis product quality control, and cannabis crop evaluation. The recent research works introduced here demonstrate an enormous potential for the application of electrochemical sensors for this purpose. The possibility of miniaturization and different modifications which allows for a better sensitivity and selectivity as well as low cost and fast response are only a few advantages of electrochemical sensors. These sensors have been proven to be sensitive enough for trace THC detection found in oral fluids after cannabis consumption. The main challenges associated with these sensors, however, are nonspecific interaction and the interference of compounds and species from the heavy plant matrix or oral fluids. Also, sensors modified with antibodies or other proteins require special storage conditions such as being refrigerated at certain temperatures. The lifetime of the bio-recognition elements used in these sensors is also limited. Development of stable and long-lasting sensors with a higher selectivity and minimum nonspecific interactions and matrix interference is now the major problem that the scientific and research community has to solve. Among all the sensors reviewed in this article, the sensor developed by (Dingqiang et al. 2016) has had the lowest limit of detection under the certain conditions that they have had.

## Data Availability

Not applicable.
