# Correction: Recent advances in electrochemical sensor technologies for THC detection—a narrative review

**DOI:** 10.1186/s42238-022-00150-z

**Published:** 2022-07-14

**Authors:** Kaveh Amini, Ali Sepehrifard, Ali Valinasabpouri, Jennifer Safruk, Davide Angelone, Tiago de Campos Lourenco

**Affiliations:** Selective Lab Inc., Richmond Hill, ON Canada

**Correction: J Cannabis Res 4, 12 (2022)**

**https://doi.org/10.1186/s42238-022-00122-3**

Following the publication of the original article (Amini et al. 2022), the author agreed to an update in the Competing Interest section and should read:

“All authors are beneficiaries of, and receive financial compensation from, Selective Lab Inc., which develops sensors for THC (https://selectivelab.com/pages/about).

Selective Lab Inc. had no role in the design and conduct of the experiments or the analysis of the data reviewed in this manuscript.”

The original article has been updated.
